# Ventricular septal rupture after acute myocardial infarction in a patient with venous thromboembolism complicated by thrombocytopenia: A case report

**DOI:** 10.1002/ccr3.7059

**Published:** 2023-03-08

**Authors:** Yuansong Zhu, Suxin Luo, Chun Zeng, Bi Huang

**Affiliations:** ^1^ Department of Cardiology The First Affiliated Hospital of Chongqing Medical University Chongqing China; ^2^ Department of Radiology The First Affiliated Hospital of Chongqing Medical University Chongqing China

**Keywords:** acute myocardial infarction, heparin‐induced thrombocytopenia, thrombocytopenia, venous thromboembolism, ventricular septal rupture

## Abstract

A woman that suffered burns previously presented with leg swelling and was diagnosed with venous thromboembolism. Heparin was given until she suddenly developed myocardial infarction. Ventricular septal rupture was detected and managed by transcatheter closure. She developed massive bleeding and extensive thrombosis that made treatment paradoxical and eventually died.

## Graphical abstract

Ventricular septal rupture was managed by transcatheter ventricular septal defect closure device in a patient with acute myocardial infarction subsequent to potential heparin‐induced thrombocytopenia.

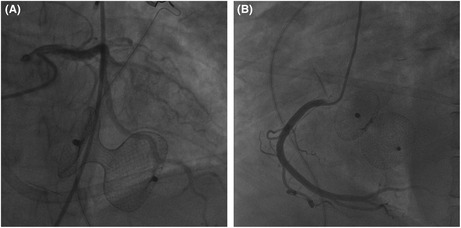

## 1 BACKGROUND

A series of devastating cardiovascular events occurred after a burn accident in a middle‐aged woman. From deep venous thrombosis (DVT) to acute myocardial infarction (AMI) to ventricular septal rupture (VSR) and the paradox between massive bleeding and extensive thrombosis, there are many pathophysiologic processes involved in the course of her disease, of which heparin‐induced thrombocytopenia (HIT) probably played the most crucial role. Despite the active management administered, this patient eventually died of multiple organ failure (MOF). In this study, we wish to report the “butterfly effect” of a burn accident and the fatal effect that HIT has played in this chain reaction.

## 2 CASE PRESENTATION

**FIGURE 1:**
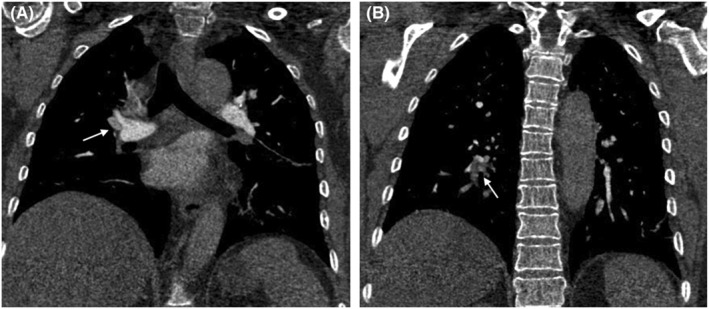
CTPA demonstrated filling defects of the right upper pulmonary artery (A) and right lower pulmonary artery (B), suggesting pulmonary embolism.

**FIGURE 2:**
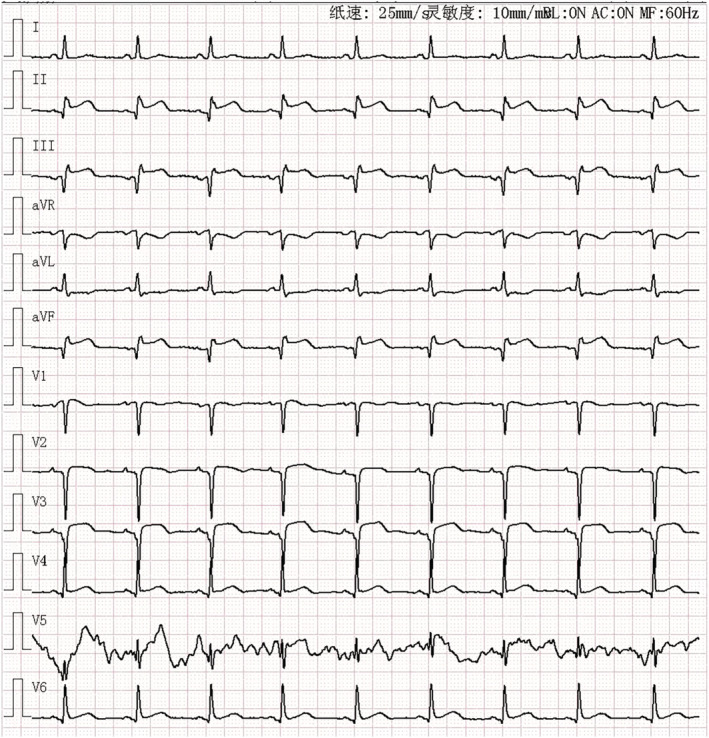
Electrocardiogram revealed ST elevation with pathological Q waves in the inferior and anterior leads, indicating acute ST elevation myocardial infarction.

**FIGURE 3:**
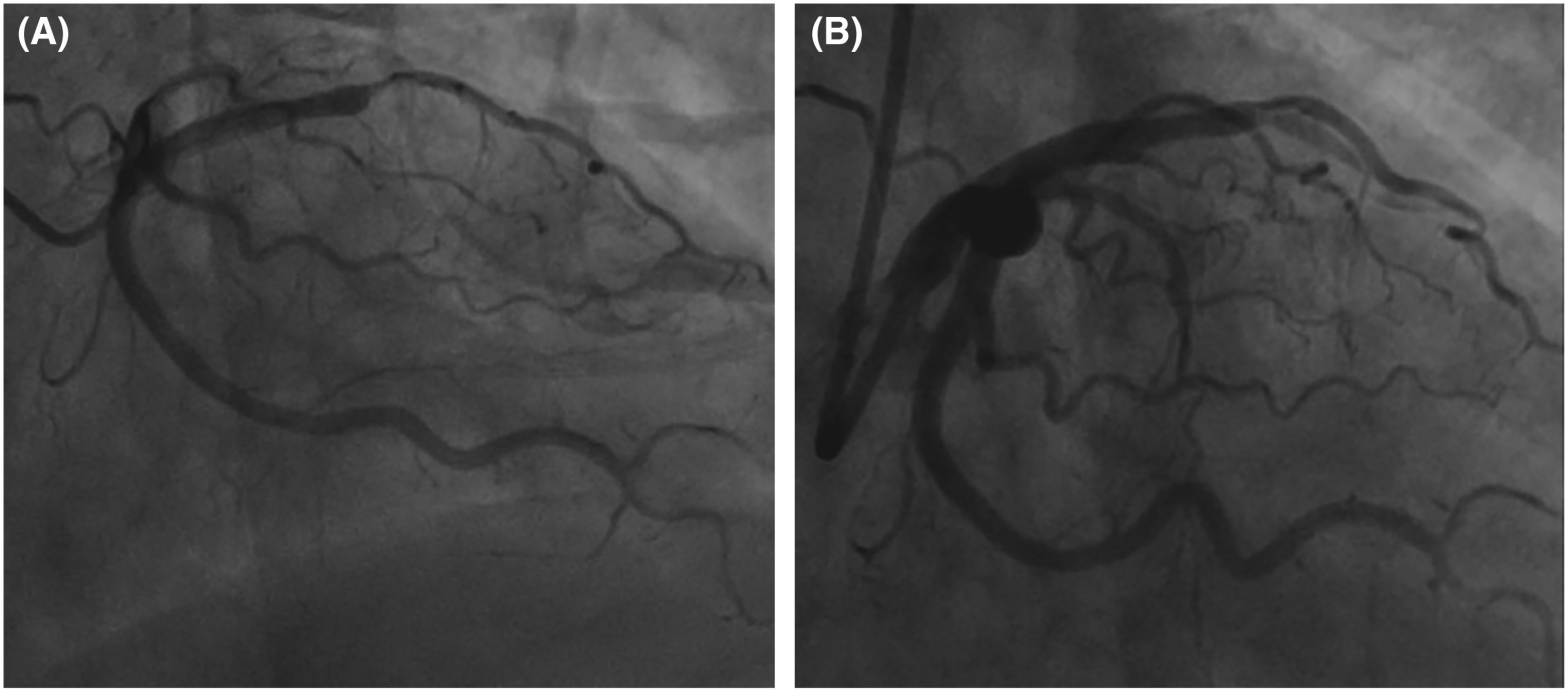
(A) Coronary angiography demonstrated a subtotal occlusion of the left anterior descending artery (LAD). (B) The percutaneous angioplasty of LAD was performed by multiple balloon dilatations, with a post‐procedure TIMI flow grade of I to II.

**FIGURE 4:**
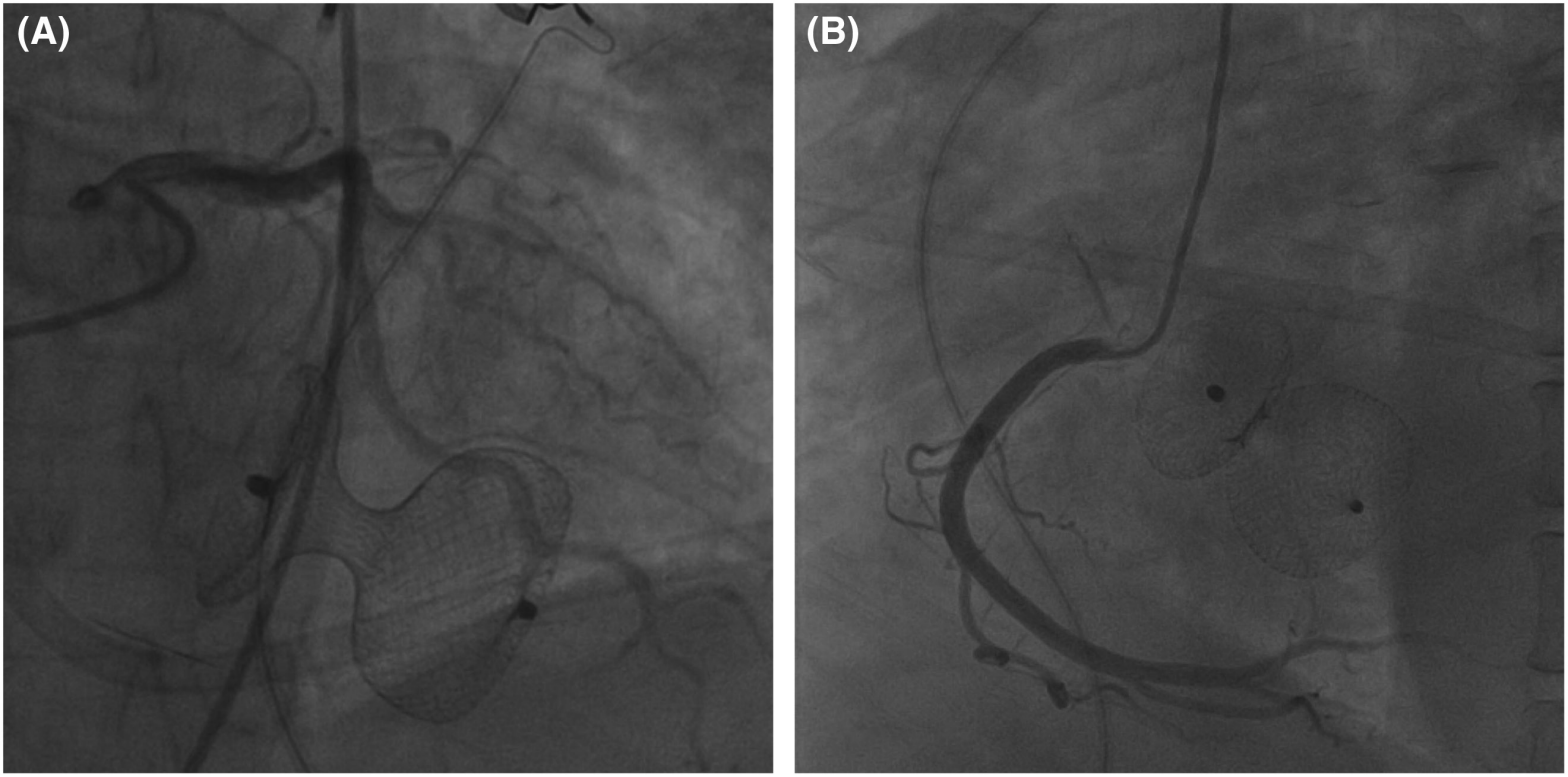
Transcatheter VSR occlusion was successfully performed using a ventricular septal defect occluder.

A 53‐year‐old woman presented to the emergency room with left leg swelling, which extended from her calf up to her thigh over the past week. Forty‐three days ago, she had suffered burns that affected her abdomen and left extremities while cooking and had been told to rest more to reduce wound friction and infection. At the local hospital, an ultrasound was performed, and DVT was found in her left lower extremity. The patient was then transferred to our hospital (Day 0, as the timeline presented in Table 1). On examination, her vitals were stable, and lungs were clear, and no cardiac murmur was detected. There were scars of burns with exudation on the left lower extremity. Both the thigh circumference and the calf circumference on the left side were increased. Complete blood count (CBC) revealed a platelet count of 197*10^9^/L. Venous ultrasound showed extensive thrombosis of the left lower extremity. Computed tomography pulmonary angiography (CTPA) later demonstrated pulmonary embolism (PE). (Figure 1) She was thus administered with subcutaneous low‐molecular weight heparin (LMWH) and intravenous tirofiban. The patient's general conditions remained stable during treatment, so the antithrombotic regimen was transitioned to oral rivaroxaban on Day 7. On Day 10, the patient reported multiple notable rashes over her trunk and extremities. Allergic reaction to rivaroxaban was suspected, so rivaroxaban was suspended and LMWH and tirofiban was resumed. The rashes gradually subsided after anti‐allergic treatment. On Day 11, the patient developed acute severe chest pain, which proved to be ST segment elevation myocardial infarction (STEMI), based on the elevated cardiac troponin I and the electrocardiogram (Figure 2). The coronary angiography revealed subtotal occlusion of the proximal left anterior descending artery (LAD), and percutaneous transluminal coronary angioplasty (PTCA) was performed (Figure 3). The antithrombotic regimen after STEMI was switched to LMWH plus a dual‐antiplatelet therapy of aspirin and clopidogrel. However, the CBC at this time showed a platelet count of 53*10^9^/L, significantly lower than admission. HIT was highly suspected after reviewing possible causes of the thrombocytopenia, with a clinical 4T score, the most widely used score for HIT screening, of 7. The LMWH was suspended, and after communication with the patient and her family, rivaroxaban plus clopidogrel was given under close monitoring. It turned out no rashes appeared during the next couple of days, and her platelet count also recovered to 314*10^9^/L. Nevertheless, on Day 20, the patient developed dyspnea and a holosystolic cardiac murmur was detected at the left sternal border. The echocardiography detected a VSR of 1.2 cm in diameter at the muscular part. Considering the weak infarcted myocardium at the acute phase, the high bleeding risk with multiple antithrombotic drugs given at the same time, and the patient's wills, selective transcatheter closure of the VSR was considered. Conservative management of medications and intra‐aortic balloon pump (IABP) was then given to bridge the patient to a possible transcatheter closure. After the placement of IABP, low dosage of heparin was used for flushing for a few days to prevent thrombosis, and then, the patient's platelet count witnessed a drop again, to 56*10^9^/L. Three weeks later, transcatheter VSR closure was performed successfully (Figure 4). Nevertheless, on Day 42, the patient developed massive gastrointestinal hemorrhage (GIH) and skin petechiae, with hemoglobin falling to 67 g/L. Meanwhile, the patient felt severe pain in her right foot, and a vascular ultrasound showed thrombosis in the right dorsalis pedis artery. The massive bleeding and extensive thrombosis then made treatment paradoxical. As the bleeding was deemed life‐threatening at this stage, all antithrombotic drugs were temporarily suspended, and blood transfusion and hemostatic agents were given. She also developed refractory heart failure during this period, despite the administration of cardiotonic agents, vasodilators, and continuous renal replacement therapy. Later, MOF and right foot gangrene ensued, and the patient eventually died at Day 51.

**TABLE 1:** Detailed timeline of this patient.

Date	Events	Antithrombotic regimen
Day ‐43	Patient suffered multiple burns when she was cooking.	
Day ‐6	Swelling in the left leg appeared and gradually aggravated.	
Day 0 (September 7)	Deep venous thrombosis was detected by ultrasound, and the patient was transferred to our hospital. Baseline platelet counts 197*10^9^/L.	
Day 1	Pulmonary embolism by CTPA; LMWH and tirofiban were given.	LMWH, tirofiban
Day 7	General condition stable and antithrombotic regimen was transitioned to oral rivaroxaban.	Rivaroxaban
Day 10	Multiple rashes were seen across the patient's trunk and extremities and allergic reaction to rivaroxaban was suspected.	LMWH, tirofiban
Day 11	Patient developed sudden chest pain which proved to be STEMI.	
Day 12	PTCA of LAD was performed and antithrombotic regimen was changed to LMWH plus a dual‐antiplatelet therapy.	LMWH, aspirin, clopidogrel
Day 12	Platelet counts decreased to 53*10^9^/L; HIT was highly suspected.	
Day 13	LMWH was suspended and rivaroxaban was given under close monitoring and no rashes appeared this time.	Rivaroxaban, clopidogrel
Day 18	The platelet counts recovered to 314*10^9^/L.	
Day 20	Patient developed dyspnea and orthopnea; VSR was detected.	
Day 33	IABP was inserted, and low dosage of heparin was used for flushing.	
Day 37	Thrombosis of right dorsalis pedis artery. Platelet counts dropped to 56*10^9^/L, again.	
Day 40	Transcatheter closure of VSR was performed.	
Day 42	Gastrointestinal bleeding and skin petechiae occurred; all antithrombotic drugs were suspended; blood transfusion and hemostatic agents were given.	All antithrombotic drugs suspended
Day 46	CRRT treatment.	
Day 48	Patient developed MOF (heart, kidney, liver, and coagulation) and right foot gangrene.	
Day 49	Patient's family requested discharge.	
Day 51	The patient died.	

Abbreviations: CRRT, continuous renal replacement therapy; CTPA, computed tomography pulmonary angiography; HIT, heparin‐induced thrombocytopenia; IABP, intra‐aortic balloon pump; LAD, left anterior descending artery; LMWH, low‐weight molecular heparin; MOF, multiple organ failure; PTCA, percutaneous transluminal coronary angioplasty; STEMI, ST elevated myocardial infarction; VSR, ventricular septal rupture.

## 3 DISCUSSION AND CONCLUSIONS

Although many pathophysiologic processes are involved in the course of her disease, HIT probably played the most crucial role. HIT is a profoundly dangerous, potentially lethal, immunologically mediated adverse drug reaction to unfractionated heparin or, less commonly, to LMWH, with a prevalence of 0.1%–5.0%.^1^ Parameters associated with higher risk of HIT development include prolonged duration of heparin therapy, unfractionated heparin over LMWH, surgical or trauma patients over medical or intensive care unit patients, and female gender.^1^, ^2^ Heparin exposure leads to the formation of antibodies that recognize complexes of platelet factor 4 and heparin, causing platelet activation, release of procoagulant microparticles,^3^ and finally the formation of thrombi, which is the clinical hallmark of HIT. There are three patterns of onset for HIT: rapid, typical, and delayed. 60% of patients exhibit the typical pattern, which manifests as a platelet decline 5 to 10 days after exposure.^1^ The AMI event and the first platelet decline of this patient is most likely to be attributed to this pattern. In 30% of cases, the onset is rapid, where platelet count declines immediately post‐exposure, as a result of previous exposure to heparin. This rapid pattern could explain the second platelet decline after heparin exposure due to IABP insertion and the lower extremity arterial thrombosis. The third pattern of delayed‐onset HIT occurred an average of 9.2 days after therapy initiation, while symptoms and signs can appear up to 3 weeks later.^4^

The diagnosis of HIT involves both clinical and laboratory components. There are several proposed scores to predict the likelihood of HIT by clinical characteristics, and the most commonly used is the 4T score by Warkentin et al.,^5^ which gives a score of 0 to 2 to each of the following categories: (1) the degree of thrombocytopenia; (2) the timing of platelet decline after heparin administration; (3) the presence of thrombosis or other HIT sequelae; and (4) the probability of other causes of thrombocytopenia. This patient had a score of 7 (2 for platelet count falls >50% and nadir ≥20*10^9^/L, 1 for onset at Day 12, 2 for new thrombotic event, and 2 for nonapparent other causes of thrombocytopenia), and thus was deemed at high risk of undergoing HIT. It is worth mentioning that tirofiban‐induced thrombocytopenia should be differentiated, but tirofiban‐induced thrombocytopenia usually occurs within a few hours after exposure and is associated with a higher risk of bleeding rather than thrombosis.^6^ The 4T score has a high negative predictive value to exclude HIT, but the positive predictive value is less reliable. Thus, laboratory testing should be prompt for further investigation, which include immunologic or functional assays to detect the immune response or activation of platelets.^1^, ^7^ Regrettably, the laboratory tests for HIT were not available in this patient, and therefore, her diagnosis of HIT was made clinically. However, some evidence has made this diagnosis more certain. First, the thrombocytopenia was detected only on Day 12 due to the negligence of platelet count monitoring but could have already happened earlier between Days 5 to 10, matching the typical pattern of HIT. Second, although the cause of the rashes the patient developed after she was transitioned to rivaroxaban was attributed to rivaroxaban, the fact that no rashes occurred after later re‐initiation of rivaroxaban has overturned this hypothesis. It now seems possible that the rashes could have been a manifestation of skin lesion from HIT, as she had been on LMWH for 6 days prior to switching to rivaroxaban.^8^, ^9^ Last, the recovery of platelet after discontinuation of LMWH and the drop again when heparin was used for flushing after IABP insertion further supported such a diagnosis.

Although venous thromboses are more common in HIT, arterial thromboses may also occur. According to a 14‐year study that analyzed 127 HIT patients, venous and arterial thrombotic events occurred in 78 and 18 patients, respectively.^10^ Among the arterial thrombotic events that have been reported in patients with HIT, AMI is less frequently seen, but its outcomes could be catastrophic. This is the first report of VSR after AMI in a patient with clinically diagnosed HIT and subsequent transcatheter closure of the VSR. VSR represents the most severe mechanical complication of AMI, with a high mortality at 41%–80%.^11^, ^12^ For VSR, immediate surgery can lead to recurrent septal defects due to the weak and friable myocardium in the acute phase and is associated with increased mortality. Delayed surgery after 3–4 weeks of medical optimization and mechanical support is considered the ideal treatment, while transcatheter device closure has also been explored as an alternative.^13^ Previous evidence for transcatheter closure of VSR was limited to case reports and case series. According to these data, the technical success could be high at 86%–93%.^14^, ^15^, ^16^, ^17^, ^18^ The in‐hospital mortality is mostly associated with MOF due to cardiogenic shock, but those who managed to discharge tended to have favorable long‐term outcomes. Although the procedure in this patient was technically successful, the patient eventually progressed to MOF, probably due to the complexity of her condition, which was characterized by refractory heart failure after VSR on the one hand, as well as massive bleeding and extensive thrombosis at the background of HIT and the usage of multiple antithrombotic drugs on the other.

With respect to the management of HIT, it is of paramount importance to terminate any source of heparin administration when a clinician has at least a moderate suspicion of HIT, including heparin, LMWH, heparin‐coated catheters, and heparin flushes.^3^, ^19^ Cessation of heparin alone is not enough to prevent thrombotic events, and rapid‐acting, alternate anticoagulation should be initiated. Two direct thrombin inhibitors lepirudin and argatroban are approved for the management of HIT, and bivalirudin is approved for use in patients with, or at risk of HIT undergoing percutaneous coronary intervention.^1^, ^7^ The usage of direct oral anticoagulants (DOACs) in HIT have also been reported but are limited to case series or studies with small sample size.^7^ Since lepirudin was no longer manufactured, and that argatroban and bivalirudin were both expensive and not covered by medical insurance in China, rivaroxaban was finally given to this patient. Rivaroxaban appeared to be safe and effective by the later recovery of platelet count and the absence of further thrombotic tendencies during this period, until low‐dose heparin was used again for flushes after IABP insertion, which was likely to have triggered the immune response to heparin for the second time.

Although currently no studies have directly addressed the benefit over risk of platelet count monitoring in patients receiving heparin or LMWH, the catastrophic outcomes following HIT of this patient have clearly warranted its necessity. The 2018 ASH clinical guidelines recommend platelet count monitoring be performed every other day from Day 4 to Day 14 (or until heparin in stopped) for patients considered at high risk (>1%) of HIT, and every 2 to 3 days for patients considered at intermediate risk (0.1%–1%) of HIT, and no platelet count monitoring for patients at low risk (<0.1%).^20^

In summary, this patient experienced a series of devastating cardiovascular events following a minor burn accident, of which the most important trigger was HIT during heparin treatment. This case has provided painful lessons to be learnt for physicians. First, the prevention of DVT in trauma patients, including burned patients, is important; second, it is necessary to monitor platelet count in patients with at least moderate risk of HIT during heparin therapy; third, any type and any dosage of heparin exposure should be avoided when HIT has already been suspected.

## AUTHOR CONTRIBUTIONS

**Yuansong Zhu:** Data curation; writing – original draft. **Suxin Luo:** Writing – review and editing. **Chun Zeng:** Visualization. **Bi Huang:** Writing – review and editing.

## FUNDING INFORMATION

Not applicable.

## CONFLICT OF INTEREST STATEMENT

The authors declare that they have no competing interests.

## ETHICS STATEMENT

The study complied with the Declaration of Helsinki and was approved by the Human Ethics Committee of the First Affiliated Hospital of Chongqing Medical University.

## CONSENT

Written informed consent was obtained from the patient's next of kin for the publication of this article.

## Data Availability

Data that support the findings of this study are available from the corresponding author upon reasonable request.
